# Fatal Mucormycosis in a Diabetic Patient: A Case Report and Review of Diagnostic Challenges

**DOI:** 10.7759/cureus.69546

**Published:** 2024-09-16

**Authors:** John Overton, Ariel Velasquez, Allison Cruse, Caitlin Noble, Robert Burrow, Poonam C Sharma, William P Berlin, Robert T Brodell, Sumit P Sontakke

**Affiliations:** 1 Medicine, University of Mississippi Medical Center, Jackson, USA; 2 Pathology, University of Mississippi Medical Center, Jackson, USA; 3 Dermatology and Pathology, University of Mississippi Medical Center, Jackson, USA; 4 Dermatology, University of Mississippi Medical Center, Jackson, USA; 5 Radiology, University of Mississippi Medical Center, Jackson, USA; 6 Medical Foundations, Ross University School of Medicine, St. Michael, BRB

**Keywords:** immunocompromised, mucormycosis, rhizopus, septic shock, zygomycosis

## Abstract

Mucormycosis (zygomycosis) is a severe and often fatal mycotic infection affecting primarily immunocompromised individuals. A 61-year-old female with type 2 diabetes mellitus and end-stage renal disease developed septic shock in association with mucormycosis. Despite antifungal treatment with liposomal amphotericin B, the patient's condition rapidly deteriorated, leading to death within 48 hours. This case underscores the aggressive nature of mucormycosis, highlighting the necessity for early diagnosis using advanced diagnostic tools and prompt treatment to improve patient outcomes.

## Introduction

Mucormycosis, also known as zygomycosis, is an aggressive and often fatal fungal infection caused by molds belonging to the order Mucorales. It is the third most frequent angioinvasive fungal infection, falling behind only candidiasis and aspergillosis [1]. According to the World Health Organization (WHO), the incidence rate of mucormycosis ranges from 0.005 to 1.7 per million population [2]. It primarily affects individuals with weakened immune systems and is seldom observed in immunocompetent individuals [1]. Immunocompromised individuals, including those with poorly controlled diabetes mellitus, severe COVID-19, malignancies, solid organ transplants, and prolonged neutropenia, are particularly susceptible to mucormycosis. The infection can manifest in five major forms: rhinocerebral, pulmonary, cutaneous, gastrointestinal, and disseminated disease, with rhinocerebral and pulmonary being the most common presentations [3].

Cutaneous mucormycosis can be either primary or secondary. Primary infection can occur in immunocompetent individuals and is caused by direct inoculation of the fungal agent through damaged skin following burns or other local skin trauma. The primary infection triggers an acute inflammatory response characterized by pus, abscess formation, skin swelling, and necrosis. These lesions initially appear red and indurated but frequently develop black eschars. Secondary cutaneous mucormycosis generally arises from hematogenous spread of the pathogen. Initially, it presents as painful, erythematous, and indurated cellulitis, which subsequently develops into an ulcer with a black eschar covering it [3]. The mortality rates for mucormycosis range from 40% to 80%, depending on the site of infection and underlying medical conditions [4].

In this report, a case of cutaneous mucormycosis in a patient with diabetes mellitus is presented. The case underscores the challenges faced even after making an early diagnosis and promptly initiating aggressive treatment. 

## Case presentation

**Figure 1:**
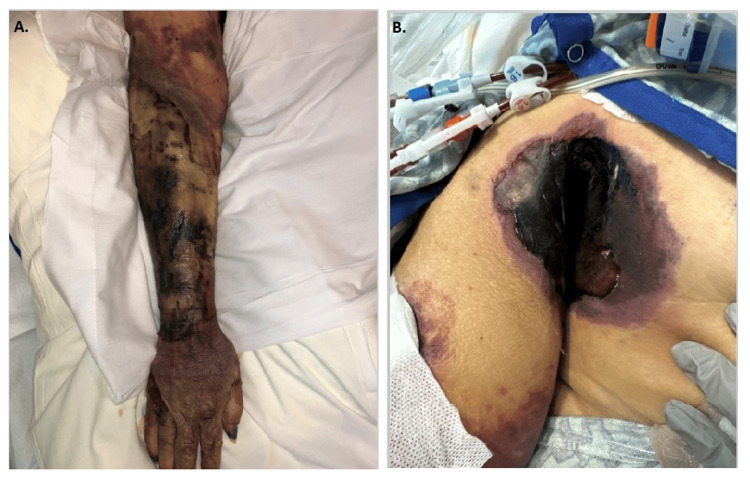
Retiform purpura and edema are noted diffusely over the right arm (A). The forearm and hand were cold to the touch. A 28 x 30 cm black eschar with a rim of purple erythema was present in the right axilla (B)

**Figure 2:**
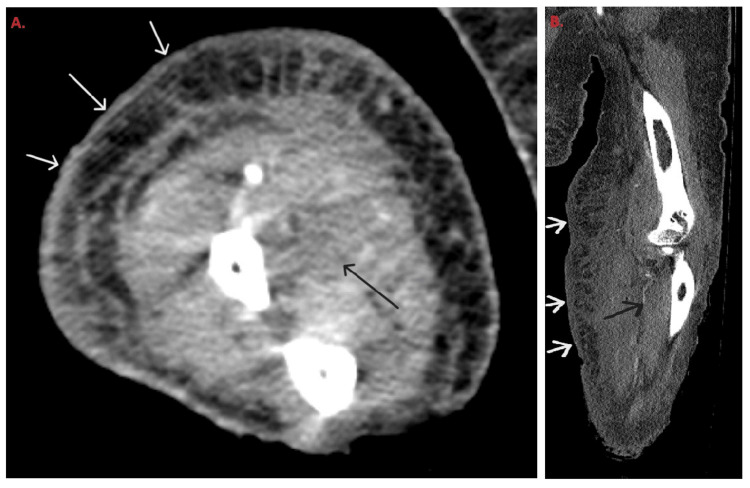
Computed tomography angiography of the upper extremity axial slice through the mid-forearm (A) and sagittal multiplanar reformat (B) demonstrate ulnar arterial occlusion (black arrows) and diffuse cutaneous irregularity (white arrows) consistent with clinically evident skin lesions

**Figure 3:**
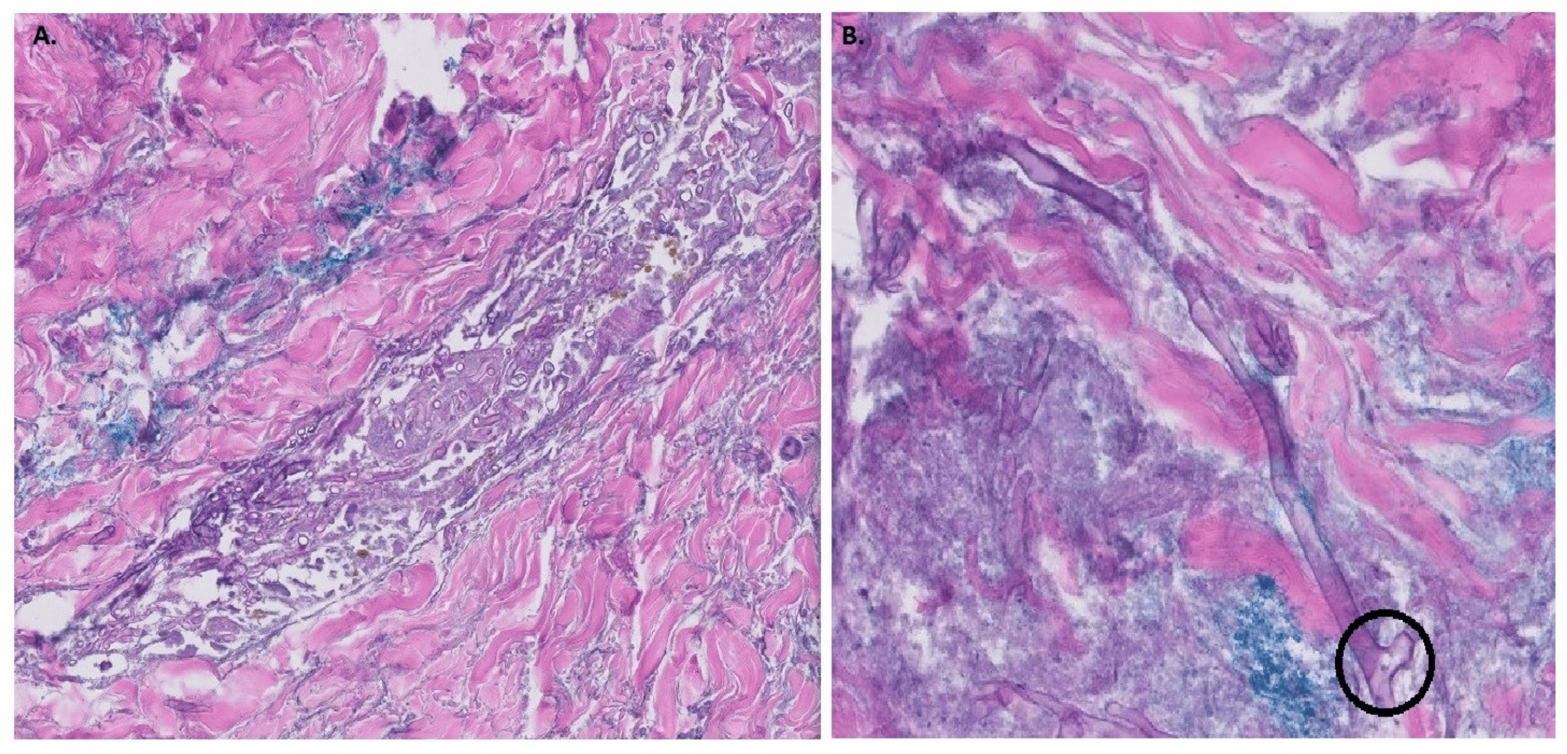
Hematoxylin and eosin-stained section showing broad ribbon like coenocytic hyphae (A) with branching (black circle) at right angles (B)

A 61-year-old female with type 2 diabetes mellitus on insulin (HbA1c-8.1%), end-stage renal disease, and severe cardiovascular dysfunction presented with hypotension secondary to septic shock with multiorgan failure. She grew vancomycin-resistant *Enterococcus* from her urine and was treated with linezolid (600 mg, Oral, two times per day) then transitioned to daptomycin (350 mg intravenously every 48 hours). *Stenotrophomonas maltophilia* grew from respiratory culture and the treatment was transitioned to levofloxacin (500 mg, every 48 hours) and minocycline (200 mg oral twice daily). A rash developed on the right arm and right axilla. The right arm showed severe generalized edema with retiform violaceous purpura, broad erosions on the forearms, and a large eschar in the right axilla (Figures 1A, 1B). Computed tomography angiography demonstrated occlusion of the right ulnar artery proximal to the ulna (Figures 2A, 2B). A punch biopsy from the right volar forearm demonstrated large, "ribbon-like" hyphae of around 6-10 µm in diameter and variable length branching at right angles within the dermis and subcutaneous fat, most notably within vascular lumina (Figures 3A, 3B). Surgical debridement was deemed unsafe. Treatment with empiric liposomal amphotericin B (10 mg/kg intravenously every 24 hours) was ineffective and the patient died within 48 hours. Meanwhile, the paraffin-embedded tissue was sent to the University of Washington clinical laboratory for the identification of the fungal species by broad-range PCR. Both 28S and Internal Transcribed Spacer sequence (ITS) rDNA PCR identified the fungus to be *Rhizopus oryzae* complex.

## Discussion

This case illustrates the aggressive nature of mucormycosis, particularly in immunocompromised patients, and underscores the complexity of its management. The patient's underlying conditions, including diabetes mellitus and end-stage renal disease, significantly heightened her vulnerability to opportunistic infection mucormycosis. The development of severe localized symptoms such as retiform purpura and eschar formation in the arm and axilla points to the angioinvasive characteristic of *Rhizopus* species, which can rapidly lead to tissue necrosis and vascular occlusion. 

The diagnostic process in this case was multifaceted, involving clinical, radiological, and histopathological evaluations. We could presumptively identify the fungus to be a Mucorales based on the morphology and branching pattern. The right-angled branching in fungi is an important morphological feature that aids in the identification and diagnosis of specific fungal infections, particularly those caused by the order Mucorales, such as *Mucor* and *Rhizopus* species. These fungi are typically characterized by broad, aseptate, ribbon-like hyphae that branch at right angles. This morphological trait is crucial for distinguishing these fungi from others, such as *Aspergillus* species, which display acute angled branching (around 45 degrees) [5]. The use of broad-range fungal PCR was crucial in accurately identifying the pathogen as *Rhizopus* *oryzae*, emphasizing the importance of advanced diagnostic tools in cases where traditional methods might fail to yield timely results. However, despite accurate identification and prompt treatment, the rapid progression of the infection highlights the challenges in managing this devastating disease.

Diagnosing invasive mucormycosis is difficult because clinical symptoms and imaging are not specific, blood cultures often reveal no growth of the organisms, and specific biomarkers are unavailable. Though conventional methods including microscopy and culture are the gold standard, they have poor sensitivity. Moreover, cultures can take weeks to produce a positive result. Consequently, alternative culture-independent tests detecting genetic material may be useful for faster diagnosis [6]. Hence, the broad-range fungal PCR was used for identifying the fungal pathogen in this case. The most common etiologic agents for mucormycosis are *Rhizopus*, *Mucor*, and *Lichtheimia* species, followed by *Rhizomucor*, *Apophysomyces*, *Cunninghamella*, and *Saksenaea* species [4]. Rhizopus oryzae is responsible for nearly 70% of all cases of mucormycosis [7].

Multidisciplinary approaches, involving infectious disease specialists, dermatologists, microbiologists, and surgical teams, are essential in managing these patients. The key principles of treatment include timely diagnosis, addressing and reversing underlying predisposing factors, prompt surgical debridement of the infected focus, and rapid initiation of effective antifungal agents systemically in high doses. Amphotericin B is the most effective agent, as *Rhizopus* species are inherently resistant to fluconazole, voriconazole, echinocandins, and 5-flucytosine. The minimum inhibitory concentrations for itraconazole, posaconazole, and terbinafine are variable [8]. The antifungal treatment alone is rarely effective without removing the infected focus surgically, leading to a mortality rate of 100% in patients with disseminated disease [9].

## Conclusions

The fatal outcome in this case reinforces the aggressive nature of mucormycosis and the critical importance of early diagnosis and prompt treatment. As the incidence of such infections may rise with increasing numbers of immunocompromised patients, enhanced vigilance and comprehensive management strategies are imperative to improve patient prognosis. Furthermore, it underscores the necessity for ongoing research into more effective antifungal therapies and early diagnostic markers to help better manage mucormycosis.
